# Radiofrequency Ablation, Cryotherapy Ablation, or Pulsed-Field Ablation to Treat Paroxysmal Atrial Fibrillation Unresponsive to Pharmacological Treatments: Interpreting Efficacy Through Reconstruction of Individual Patient Data From Randomized Trials

**DOI:** 10.7759/cureus.65113

**Published:** 2024-07-22

**Authors:** Melania Rivano, Luca Cancanelli, Roberto Brunoro, Chiara Nunzia Fasano Celentano, Lorenzo Di Spazio, Daniele Mengato, Andrea Messori

**Affiliations:** 1 Hospital Pharmacy, Binaghi Hospital, Cagliari, ITA; 2 Hospital Pharmacy Department, Azienda Ulss 2 Marca Trevigiana, Treviso, ITA; 3 Department of Pharmacy, University of Milano, Milano, ITA; 4 Hospital Pharmacy Department, Azienda Ulss 3 Serenissima, Mirano, ITA; 5 Hospital Pharmacy Department, Santa Chiara Trento Hospital, Trento, ITA; 6 Hospital Pharmacy Department, Azienda Ospedale Università di Padova, Padova, ITA; 7 Health Technology Assessment (HTA) Unit, Regione Toscana, Firenze, ITA

**Keywords:** indirect comparison, paroxysmal atrial fibrillation, pulse-field ablation, radiofrequency ablation, cryoballon ablation

## Abstract

Three techniques of catheter ablation (CA; radiofrequency, cryoballoon, and pulsed-field ablation) are available to treat patients with paroxysmal atrial fibrillation (PAF) who do not adequately respond to pharmacological treatments. Our study was aimed at comparing these techniques based on the data of randomized studies because these are considered the best sources of efficacy data.

After selecting pertinent trials, our analysis studied the time-to-event data published for these three techniques. An artificial intelligence method was used that reconstructs individual patient data from the Kaplan-Meier curves. The endpoint was an arrhythmia recurrence. A preliminary heterogeneity analysis was performed. Then, our main analysis was based on individual patient data reconstructed from Kaplan-Meier graphs. The hazard ratio (HR) was its main parameter.

Three randomized trials were included. Our heterogeneity analysis confirmed an acceptable level of between-trial heterogeneity that allowed us to pool the curves from the different trials; however, cryoballoon ablation with a two-minute duration fared worse than the other techniques. Then, our main analysis estimated the following values of HR: pulsed-field ablation versus radiofrequency ablation, 0.549 (95%CI, 0.413-0.730; p<0.001); pulsed-field ablation versus cryoballoon ablation, 0.478 (95%CI, 0.364-0.633); radiofrequency ablation versus cryoballoon ablation, HR=0.871 (95%CI, 0.585-1.295; p=0.506).

In conclusion, radiofrequency ablation and cryoballoon ablation showed similar effectiveness (except for the two-minute cryoballoon ablation, which fared worse). Our results showing the superiority of pulsed-field ablation versus thermal ablation must be interpreted with caution because the patients given pulsed-field ablation were limited, and their follow-up was shorter than that of patients receiving thermal ablation.

## Introduction and background

Atrial fibrillation is the most common cardiac arrhythmia and is associated with an increased risk of stroke, heart failure, and sudden death [1]. Patients experiencing short episodes of atrial fibrillation that terminate spontaneously or with intervention within seven days are classified as having paroxysmal atrial fibrillation (PAF). Anti-arrhythmic medications and catheter ablation (CA) are the most important heart rhythm control treatment options for these patients. These treatments have the potential to affect mortality and morbidity outcomes, although anti-arrhythmic drugs generally have limited efficacy [2-5]. Regarding the differences between PAF and persistent atrial fibrillation, these two conditions are evaluated separately in most clinical guidelines [2,3] because they differ considerably in a large number of aspects regarding symptoms, diagnosis, treatment options, outcome parameters, etc.

CA is the primary curative therapy for several arrhythmias. It uses catheters to locate the region of the heart from which arrhythmias are generated and then ablates it to prevent continued electrical disruption.

The literature on the effectiveness of CA to treat PAF unresponsive to medical treatment continues to evolve [6-8]. In the past decade, a few randomized trials have investigated two types of thermal ablation (radiofrequency or cryoballoon) as first-line treatments for this disease condition [9-11].

Radiofrequency ablation is the targeted cautery of cardiac tissue by local application of radiofrequency energy and is associated with thermal damage and the deposition of energy. Once a target zone is identified during an electrophysiological study, ablation is performed by heating the interface between the catheter and endocardium until cell death occurs at temperatures over 47 °C. Balloon catheters can ablate larger areas of tissue than traditional, single-tip catheters that require point-by-point ablation. Hence, balloon-mediated CA techniques such as cryoballoon ablation have increasingly been used in the last few years. The efficacy of these interventions is generally determined based on a primary endpoint represented by arrhythmia recurrence over time.

More recently, pulsed-field ablation was proposed as a further option. This technique uses electrical pulses to cause nonthermal irreversible electroporation and induce cardiac cell death, and it may have effectiveness comparable to traditional catheter ablation while preventing thermally mediated complications. At the end of 2023, a large-scale randomized trial was published showing that pulsed-field ablation was non-inferior to thermal ablation (i.e., the ablation based on radiofrequency or cryoballoon) [12]. This study can be considered the pivotal trial for this new technique. Considering that, apart from medical therapy, three valid therapeutic options are now available for these patients (radiofrequency ablation, cryoballoon ablation, and pulsed-field ablation), a systematic analysis of all randomized studies conducted in this area is worthwhile. In this framework, the present study was undertaken with two main objectives: (i) to provide a systematic overview of the overall evidence of efficacy deriving from these randomized trials; and (ii) to improve the methodological value of this overall analysis by applying an artificial intelligence technique (the IPDfromKM method [13-16]) that reconstructs individual patient data from the published graphs of Kaplan-Meier curves. Although our study is similar to the two meta-analyses recently published by Aldaas et al. [17] and Zhang et al. [18], the use of the IPDfromKM method represents an interesting methodological advantage that characterizes our analysis.

## Review

Methods

We conducted a PubMed literature search (last query run on June 12, 2024) to identify randomized controlled trials (RCTs) eligible for this analysis. A search term “atrial AND fibrillation AND (radiofrequency OR cryo* OR pulsed*)” was employed in combination with the filter “randomized controlled trials." Results were reported according to the Preferred Reporting Items for Systematic Review and Meta-Analyses (PRISMA) statement [19]. We also searched the Cochrane Library for any recent systematic reviews on this subject, the ClinicalTrials.gov database, and the websites of the European Medicines Agency and the U.S. Food and Drug Administration. Studies written in languages other than English were not considered. The keyword “paroxysmal atrial fibrillation” was also employed for these additional searches. Our analysis included the eligible trials that met the following criteria: (a) previously treated patients with PAF; (b) evaluation of at least one treatment involving radiofrequency, cryoablation, or pulsed-field ablation; (c) determination of atrial fibrillation recurrence using a Kaplan-Meier curve with a follow-up of at least 12 months. The endpoint of our analysis was an arrhythmia recurrence. No specific thresholds for follow-up beyond 12 months were applied. No restrictions were employed in terms of the physical fitness or age of the study population. Randomized studies reported in duplicate publications were included only once. For each trial, we extracted the basic information needed for our analysis. Information on disease conditions at baseline was also recorded.

In the analysis of each treatment arm of each trial, patient-level data were reconstructed from the Kaplan-Meier curve using the IPDfromKM method. This method uses an artificial intelligence algorithm to examine the Kaplan-Meier time-to-event curves to generate reconstructed individual patient data reliably. The IPDfromKM method was run through its web-based version; all the remaining statistical analyses were performed according to specific packages designed for the R platform (RStudio, Boston, MA) [20]. Heterogeneity was assessed through the likelihood ratio test and Wald’s test. Results were presented using hazard ratios (HR) and medians along with their 95% confidence intervals (CI).

Articles selected through our literature search

**Figure 1:**
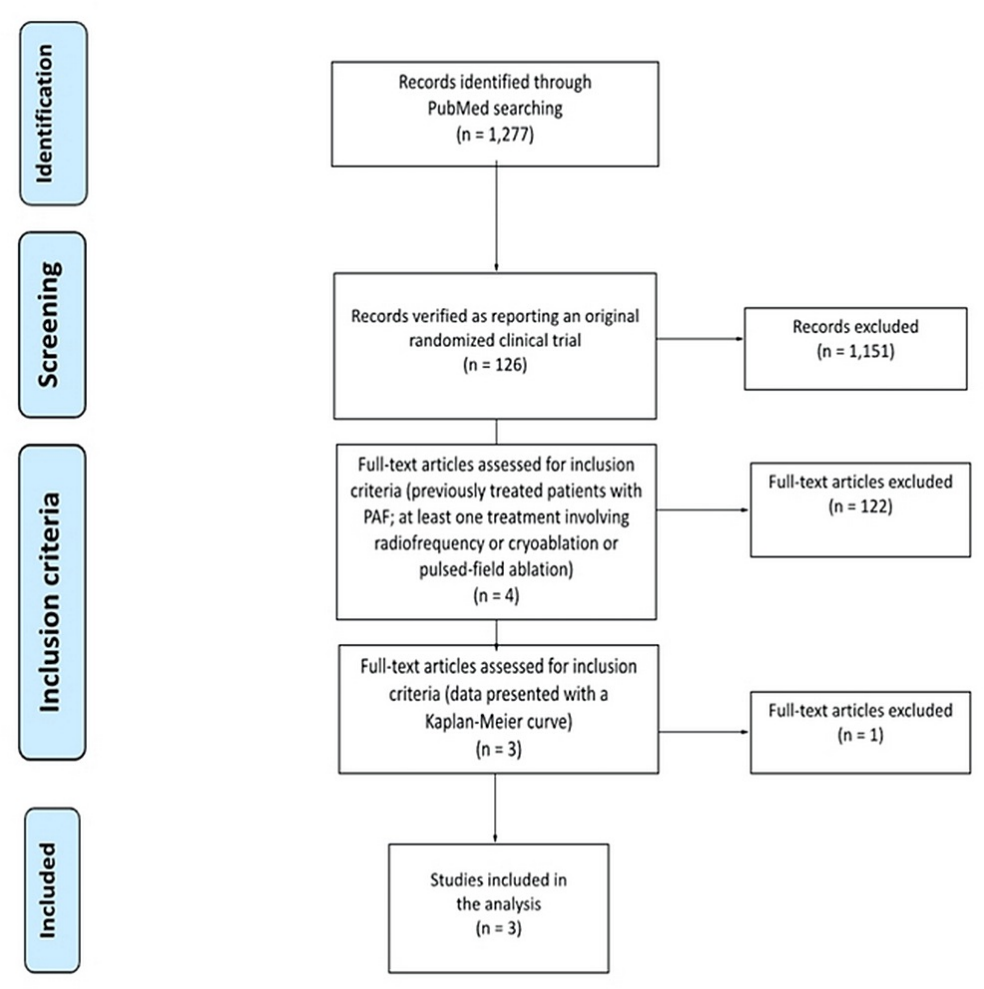
Preferred Reporting Items for Systematic Reviews and Meta-analyses (PRISMA) flow diagram. The keywords employed in our PubMed search were the following: “(atrial AND fibrillation AND (radiofrequency OR cryo* OR pulsed*)” was employed in combination with the filter “randomized controlled trials.”

Our literature search extracted 126 RCTs, from which we identified 4 eligible RCTs [9-12] (Figure 1). One (Kuniss et al. [10]) was excluded because no Kaplan-Meier curve was reported, whereas the remaining three [9,11,12] met our inclusion criteria and were included in our analysis. The type of atrial fibrillation was paroxysmal in all three studies.

Since two of these trials included a separate subgroup with persistent atrial fibrillation, these patients were excluded from our analysis (and also from Table 1). In the trial by Reddy et al. [12], the study arm of patients treated with thermal ablation was split into two subgroups given radiofrequency or cryoablation, and so the trial reported the information needed for our analysis separately for these two subgroups.

**Table 1:** Characteristics of the three included studies. *t, duration of follow-up; ^§^Clinical failure was defined as documented recurrence of atrial fibrillation, documented occurrence of atrial flutter or atrial tachycardia, prescription of antiarrhythmic drugs (class I or III), or repeat ablation; ^§§^In these two cases, the number of events was not explicitly reported in the original article; therefore, these two values 56 and 46 events were estimated by the IPDFROMKM program through a specific option of the software.

Data-set	Study design	Primary efficacy endpoint	Cohort	Inclusion criteria	Mean age (yrs) and gender	t* (months)	No. of patients	No. of recurrences
Kuck et al. [11]	Multicenter (16 centers)	Time to the first documented clinical failure^§^ occurring more than 90 days after the index ablation procedure	Radiofrequency	Pre-treated (refractory to at least one antiarrhythmic drug class I or III or beta-blockers)	60.1 (male 63%)	32.9	376	143
			Cryoballoon		59.9 (male 59%)		374	138
Reddy et al. [12]	Multicenter (30 centers)	Time to the first occurrence of a composite of initial procedural failure, documented atrial tachyarrhythmia, antiarrhythmic drug use, cardioversion, or repeat ablation	Pulsed-field ablation	Pre-treated (refractory to at least one antiarrhythmic drug class I, II, III, or IV)	62.4 (male, 66.2%)	12	301	97
			Radiofrequency		62.5 (male, 64.6%)		164	56^§§^
			Cryoballon				132	46^§§^
Andrade et al. [9]	Multicenter (8 centers)	Time to the first occurrence of persistent atrial tachyarrhythmia, as defined as the first occurrence of a continuous atrial tachyarrhythmia episode lasting 7 days or longer in duration.	Radiofrequency	Pre-treated (refractory to at least one antiarrhythmic drug class I or III)	60 (male, 68.7%)	36	115	65
			Cryoablation 2-min		61 (male, 61.2%)		116	73
			Cryoablation 4-min		59 (male, 70.4%)		115	62

Table 1 shows the main characteristics of the three trials included in our analysis. Overall, three study arms employed radiofrequency ablation; a single arm was available for pulsed-field ablation. Radiofrequency played the role of a common comparator because it was present in each of the three studies. The three trials showed some differences in their primary efficacy endpoint, but these differences do not determine any substantial impact.

Heterogeneity assessment

**Figure 2:**
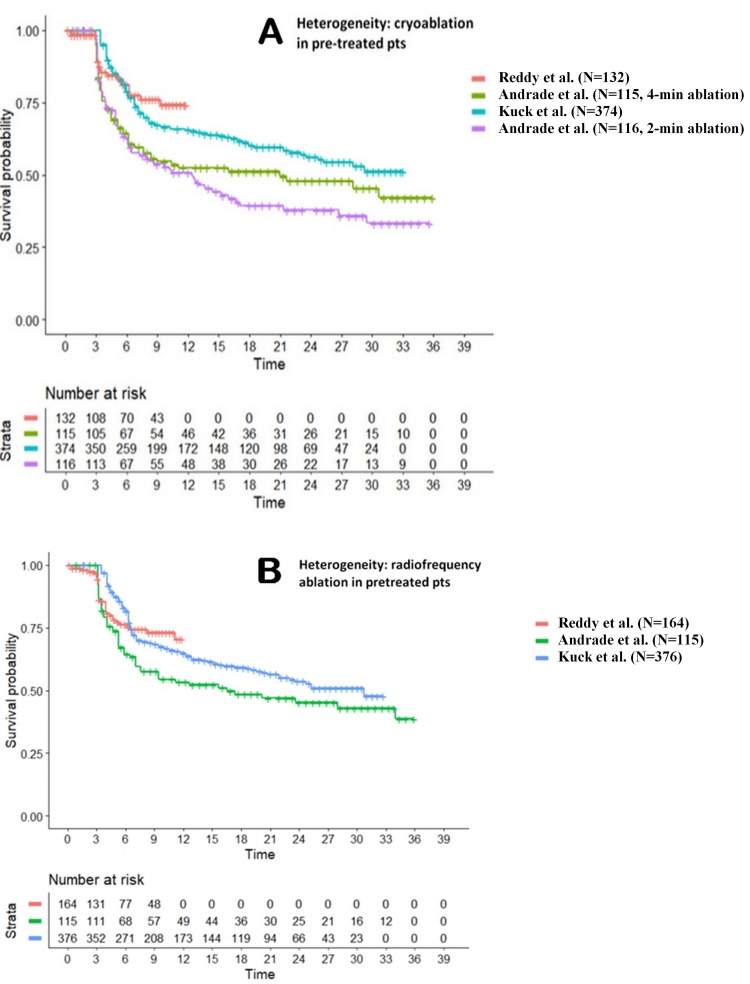
Analysis of the seven patient arms reported in the three included trials: assessment of heterogeneity. Panel A: cryoablation, 4 study arms. Panel B: radiofrequency ablation, 3 study arms. Legends: Panel A: Reddy et al. (N=132) in red [12]; Kuck et al. (N=374) in blue [11]; Andrade et al. (N=115, 4-min ablation) in green [9]; Andrade et al. (N=116, 2-min ablation) in purple [9]. Panel B: Reddy et al. (N=164) in red [12]; Kuck et al. (N=376) in blue [11]; Andrade et al. (N=115) in green [9]. Endpoint, arrhythmia recurrence. Time, in months.

Figure 2 shows our assessment of between-trial heterogeneity, which was performed separately for cryoablation (Panel A, four study arms) and radiofrequency ablation (Panel B, three study arms). No heterogeneity assessment was done for pulsed-field ablation because its clinical material included just a single arm. The results of our heterogeneity analysis showed that a significant level was found for cryoablation but not for radiofrequency.

The detailed results of this analysis were the following: cryoablation: likelihood ratio test = 19.19 on 3 df, p<0.001; Wald test = 19.9 on 3 df, p<0.001; radiofrequency ablation: likelihood ratio test = 5.18 on 2 df, p=0.07; Wald test = 5.52 on 2 df, p=0.06. The high heterogeneity found for cryoablation likely depends on the poor effectiveness determined by the two-minute duration option. Interestingly enough, the three study arms employing radiofrequency did not show any significant cross-trial heterogeneity. This finding supports the reliability of our analysis, at least as far as the radiofrequency option is concerned.

Main analysis with the generation of time-to-event curves based on pooled treatment groups

**Figure 3:**
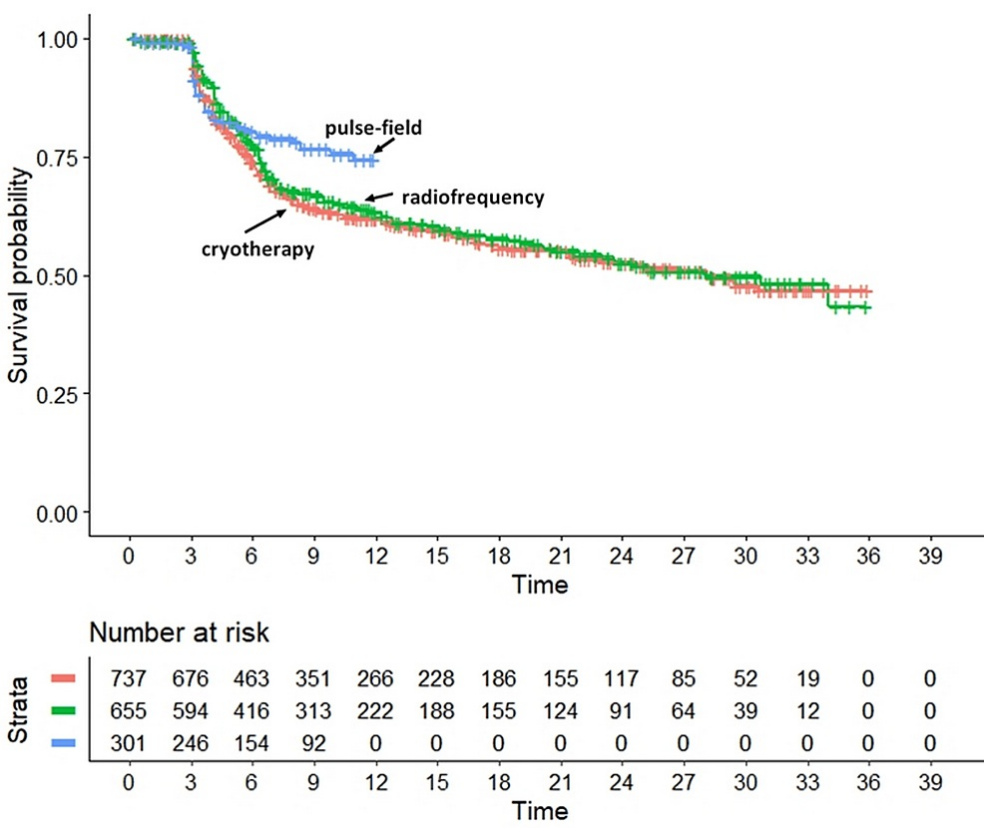
Main analysis: application of the IPDfromKM method. In red, patients undergoing cryotherapy (n=737); in blue: patients undergoing pulsed-field ablation (n=301), in green patients treated with radiofrequency (n= 655). Endpoint, arrhythmia recurrence. Time, in months.

After performing the two heterogeneity analyses, our main analysis compared the effectiveness of the three ablation techniques (Figure 3).

According to the results of the three head-to-head comparisons, the HR for pulsed-field ablation versus radiofrequency ablation was 1.82 (95% CI, 1.37 to 2.42; p<0.001), demonstrating the superiority of pulsed-field ablation. Similarly, the HR for pulsed-field ablation versus cryoballoon ablation was 2.09 (95% CI, 1.58 to 2.75; p<0.001), demonstrating the superiority of pulsed-field ablation versus cryoballoon ablation. In contrast, the comparison between radiofrequency ablation and cryoballoon ablation showed no significant difference (0.871; 95% CI, 0.585 to 1.295; p=0.506) without a clear trend in favor of either treatment.

Interpretation of findings

As previously pointed out, our investigation adopted a quite new evidence-based method (the IPDfromKM method) that reconstructs individual patient data through an artificial intelligence algorithm. This algorithm was developed to extract as much information as possible from the Kaplan-Meier graphs published in the original trials. Recent studies, especially in the areas of oncology [15] and cardiology [16], have clearly stressed the methodological advantages of this evidence-based method.

The results of our main analysis clearly indicated that radiofrequency and cryotherapy do not differ in efficacy from one another; by contrast, our finding regarding pulse-field ablation is more controversial because this form of ablation proved to be more effective than both radiofrequency and cryoablation. However, this demonstration is quite weak because pulse-field ablation included relatively few patients (belonging to a single arm of a single study) and because its length of follow-up was much shorter than that of radiofrequency and cryoablation. While an advantage of our study is the accurate management of survival data resulting from the use of the IPDfromKM method, an important limitation is that it focused exclusively on efficacy. For other clinical endpoints not strictly related to efficacy, the meta-analysis by Aldaas et al. [17] is a source of relevant information. From the analysis of six comparative studies involving a total of 1,012 patients, the main conclusion of Aldaas et al. was that pulse-field ablation is associated with shorter procedural times and longer fluoroscopy times, but there is no difference in periprocedural complications or rates of recurrent atrial fibrillation compared with thermal ablation. These results of Aldaas et al. are consistent with those recently published by Zhang et al. [18], whose meta-analysis based on 15 studies compared pulse-field ablation and cryoablation for safety and procedural efficiency; the main safety endpoints included periprocedural complications, procedure time, and fluoroscopy time. The conclusions of Zhang et al. [18] are in favor of pulse-field ablation, as it was shown to be a safer, time-saving, and tissue-specific procedure compared to cryoablation, with comparable success rates; this has the potential to improve procedural efficiency and optimize resource utilization in clinical practice.

Limitations

The limitations inherent in indirect comparisons are well known [15] and are well illustrated by this study. In particular, our comparison included radiofrequency ablation studies published in 2016, which are considerably older than the cryoballoon ablation study published in 2024 and the pulsed-field ablation study published in 2023; this difference may have some impact on our results due to the advances in the management of these patients in recent years. Of the three ablation techniques studied in our paper, pulsed-field ablation is the one that has attracted the most interest, mainly because it is much more recently developed than radiofrequency and cryotherapy, which have been available for many years. The novelty of pulsed-field ablation has stimulated much research in recent years. An interesting point is that pulsed-field ablation devices are currently manufactured by three different companies (Boston Scientific, Medtronic, and Johnson). This has led to studies comparing these three devices [21]. More recently, another important issue has arisen because the recurrent atrial arrhythmia burden has been proposed as a new parameter to better monitor the outcome of patients undergoing pulse field ablation [22]. Finally, among the limitations of our study, it should be noted that the included studies were not formally evaluated for their methodological quality; however, all three studies were published in very authoritative journals.

## Conclusions

The main finding of this study is represented by our main analysis, which compares thermal ablation and pulsed-field ablation for the treatment of PAF refractory to pharmacological therapy. From a methodological point of view, a strength of our analysis stems from the use of the IPDfromKM method, which takes into account the timing of events and presents its results in the form of Kaplan-Meier curves based on "reconstructed" patients. The superiority of pulsed-field ablation found in our study must be interpreted with caution due to the small number of patients studied to date and the lack of long-term information on recurrence rates. Although pulsed-field ablation showed superiority in efficacy, this finding must be tempered by the limitations mentioned above. In conclusion, our results have the potential to influence treatment decisions or future research directions in the field of atrial fibrillation management. Larger studies with longer follow-ups (especially for pulsed-field ablation) may be needed to validate the current findings.
